# Physicochemical, Structural, and Biological Properties of Polysaccharides from Dandelion

**DOI:** 10.3390/molecules24081485

**Published:** 2019-04-15

**Authors:** Huijing Guo, Weida Zhang, Ying Jiang, Hai Wang, Guogang Chen, Minrui Guo

**Affiliations:** College of Food Science, Shihezi University, Shihezi 832000, China; ghjshzu@163.com (H.G.); zwd9411@163.com (W.Z.); 715jy@sohu.com (Y.J.); wanghai948@126.com (H.W.)

**Keywords:** dandelion, polysaccharide, structural characterization, bioactivities

## Abstract

The edible and medicinal perennial herb dandelion is known to have antitumor, antioxidant, and anticomplement properties. However, the structural characterization and biological effects of its polysaccharides are not well understood. Here, we aimed to extract and investigate a novel polysaccharide from dandelion. A water-soluble polysaccharide, PD1-1, was successfully obtained from dandelion through ultrasonic-assisted extraction and purification using diethylaminoethyl (DEAE)–Sepharose fast flow and Sephadex G-75 columns. The results showed that PD1-1 is an inulin-type polysaccharide with a molecular weight of 2.6 kDa and is composed of glucose (52.39%), and mannose (45.41%). Glycosidic linkage analysis demonstrated that PD1-1 contains terminal α-d-Man/Glcp-(1→ and →1)-β-d-Man/Glcf-(2→ glycosidic linkage conformations. A physicochemical analysis indicated that PD1-1 has a triple helix structure and exhibits important properties, including good swelling, water-holding, and oil-holding capacities. Furthermore, PD1-1 showed good antioxidant activities in DPPH and hydroxyl free radical scavenging abilities, with IC_50_ values of 0.23 mg/mL and 0.25 mg/mL, respectively, and good hypoglycemic activities in α-amylase and α-glucosidase inhibition, with IC_50_ values of 0.53 mg/mL and 0.40 mg/mL, respectively, in a concentration-dependent manner. Results suggest that PD1-1 possesses efficacious antioxidant and hypoglycemic properties and has potential applications as a functional food ingredient.

## 1. Introduction

Polysaccharides are essential functional substances composed of various monosaccharide sugars and are usually found in animals, plants, and microorganisms [1]. Polysaccharides play an important role in the growth and development of organisms [2,3]. Plant polysaccharides are also known to possess multiple biological activities, including antioxidant [4], antitumor [5], anti-hyperglycemic [6], and immune regulation [7] activities, which are closely related to their physicochemical properties. Natural polysaccharides are suitable potential candidates for preventing and treating diseases due to their nontoxicity and negligible side effects compared with synthetic medicines [8,9]. Therefore, much research has been devoted to the discovery and investigation of natural polysaccharides.

Dandelion (*Taraxacum mongolicum*), a perennial herbaceous plant belonging to the Asteraceae family, is widely distributed in the northern regions of China, including Gansu, Shanxi, and Xinjiang [10]. Dandelion is known for its applications in the production of drinks, liquor, and toiletries, for its identified pharmacological functions, including detoxification and diuresis, and its antibacterial and antitumor properties, which have resulted in its frequent use in traditional Chinese medicine [11,12]. Dandelion roots, leaves, and flowers contain many pharmaceutical ingredients, such as phenolics, sterols, flavonoids, and polysaccharides [13]. Polysaccharides from dandelion (PD) have also been reported to regulate metabolism and pathological processes owing to anticomplement, antitumor, antioxidant, and antibacterial effects [14,15,16]. Many PD extraction methods have been investigated, including hot water extraction, enzyme extraction, microwave-assisted extraction, and ultrasound-assisted extraction [17]. However, the separation, purification, and exhaustive structural characterization of PD have been lacking. Therefore, efficient and accurate analytical methods for elucidating PD structures are required.

The present study aimed to separate and purify polysaccharides, and explore the structures and functions of the purified polysaccharide fraction. An ultrasound-assisted method was used to extract polysaccharides from dandelion from Xinjiang province. Purified fractions of dandelion polysaccharides were obtained by chromatography through diethylaminoethyl (DEAE)–Sepharose fast flow columns and then Sephadex G-75 columns. The physicochemical properties and structures of the purified fractions were determined using Fourier transform infrared (FT–IR) spectroscopy, gas chromatography–mass spectrometry (GC–MS), nuclear magnetic resonance (NMR) spectroscopy, colorimetric determination with Congo red, and high performance gel permeation chromatography (HPGPC). Moreover, the in vitro antioxidant and hypoglycemic activities of the purified polysaccharide were evaluated by the assay of free radical scavenging activities and enzyme inhibitory activities. This strategy could be promising for application to novel functional foods or drugs with polysaccharides as ingredients.

## 2. Results and Discussion

### 2.1. Polysaccharide Isolation and Purification

**Figure 1:**
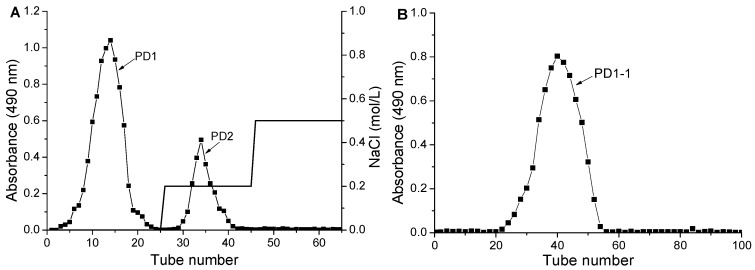
Chromatograms from (**A**) Diethylaminoethyl (DEAE) cellulose fast flow anion-exchange column chromatography of crude polysaccharides, and (**B**) Sephadex G-75 column chromatography of PD1.

Crude polysaccharide was separated on a DEAE–Sepharose fast flow anion-exchange column, eluting with distilled water and the stepwise addition of different NaCl solutions (0.2, 0.5, 1.0, and 2.0 mol/L). According to the elution curve (Figure 1A), two polysaccharides were separated and designated as PD1 and PD2. The first fraction, PD1, was eluted by distilled water, while the second, PD2, was eluted by 0.2 mol/L NaCl. The pooled polysaccharide fraction (PD1) was then collected, concentrated, dialyzed, lyophilized, and further purified through a Sephadex G-75 gel filtration column. As shown in Figure 1B, one fraction (PD1-1) was eluted by distilled water. PD1-1 was collected and used in the following experiments.

### 2.2. Chemical Analysis of PD1-1

The chemical composition of PD1-1 was analyzed. Polysaccharide PD1-1 mainly contained 95.35 ± 3.45% total sugars, 6.61 ± 0.22% uronic acid, and no protein, as determined by the Coomassie Brilliant Blue G-250 method producing no response. The molecular weight of PD1-1 was determined to be 2.6 kDa by high performance gel permeation chromatography (HPGPC), based on the calibration curve that had been established using dextran standards (Figure S1). Monosaccharide composition analysis showed glucose (52.39%) was the major sugar in PD1-1, along with a considerable amount of mannose (45.41%). The major monosaccharides, glucose and mannose, had a molar ratio of approximately 1:1 (Figure S2), thus it was speculated that PD1-1 is a fructan, because fructose is a ketose, which is reduced to mannose and glucose when analyzed by GC-MS.

### 2.3. Structural analysis of PD1-1

#### 2.3.1. UV and FT-IR Spectrum Analysis

**Figure 2:**
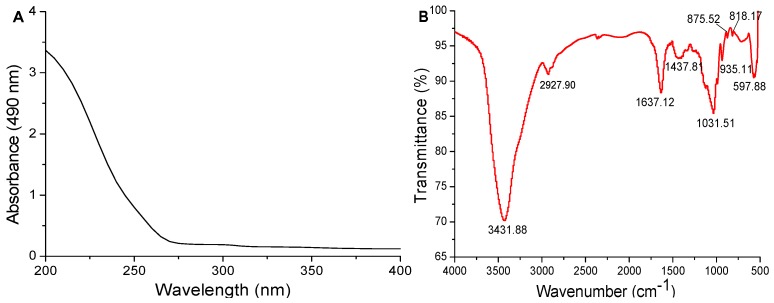
(**A**) UV and (**B**) FT-IR spectra of PD1-1.

The ultraviolet–visible (UV–vis) spectrum of PD1-1 is shown in Figure 2A. PD1-1 showed no absorption at 260 and 280 nm, indicating the absence of protein. This was consistent with results of Bradford’s method. The FT-IR spectrum of PD1-1 was scanned in the range of 400–4000 cm^−1^, exhibiting a typical polysaccharide absorption peak. As shown in Figure 2B, the strong peak at 3431 cm^−1^ was attributed to O–H group stretching vibrations, the peak at around 2927 cm^−1^ was attributed to C–H group stretching vibrations, bands at 1637 and 1437 cm^−1^ were attributed to flexural vibrations of C=O groups, and the signal at 1031 cm^−1^ was attributed to C–O group stretching vibrations. The characteristic peaks at 935, 875, and 818 cm^−1^ were attributed to fructose with α-type and β-type glycosidic linkages simultaneously [18,19]. These results confirmed that PD1-1 showed typical polysaccharide absorption peaks.

#### 2.3.2. Methylation Analysis

After methylation of PD1-1, the methylated derivatives were analyzed using GC–MS. As shown in Table 1, the results showed that PD1-1 had two glycosidic linkages, namely 2,3,4,6-Me_4_-Glc/Manp and 3,4,6-Me_3_-Glc/Manf linkages, with contents of 2 and 14 mol%, respectively. These results show that PD1-1 mainly contains α-d-Glc/Manp-1- and -1-β-d-Glc/Manf-2- glycosidic bonds [20].

#### 2.3.3. NMR Analysis

**Figure 3:**
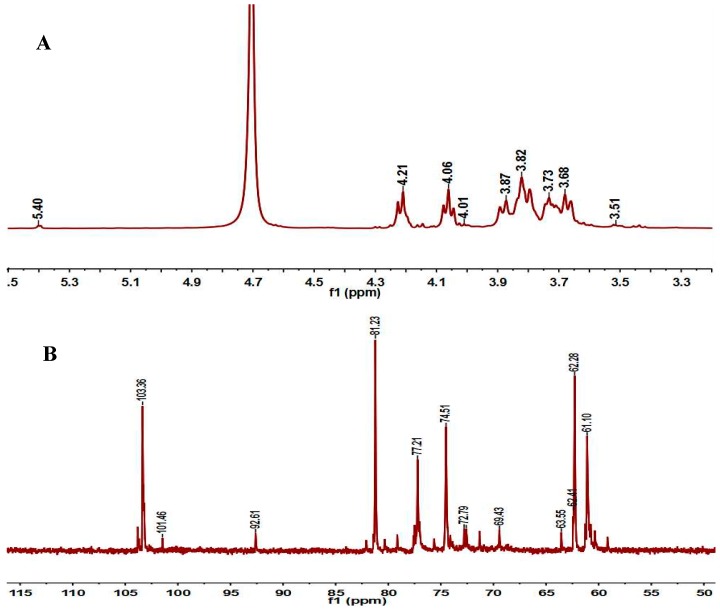

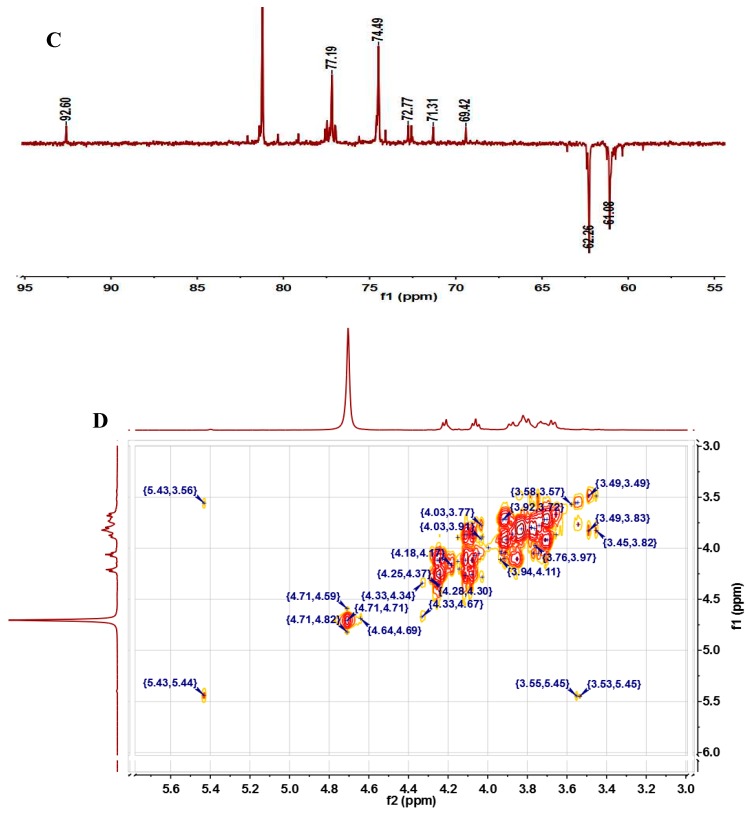

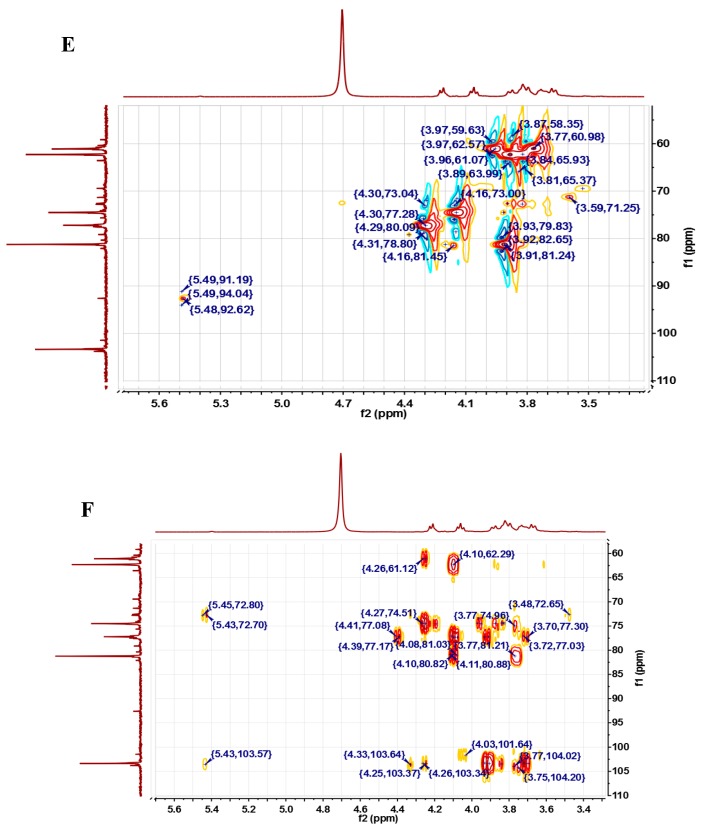
NMR spectra of PD1-1: (**A**) 1H; (**B**) 13C; (**C**) DEPT135; (**D**) 1H–1H COSY; (**E**) HSQC; and (**F**) HMBC.

The structure of PD1-1 was further analyzed using 1D and 2D nuclear magnetic resonance (NMR) spectroscopy. The major ^1^H and ^13^C-NMR chemical shifts for PD1-1 are shown in Table 2. The ^1^H-NMR spectrum (Figure 3A) showed one anomeric H-1 signal at δ 5.4 ppm, which indicated that the glucose was an α-d-Glcp residue corresponding to the C-1 signal at 92.61 ppm in the ^13^C-NMR spectrum and other proton resonances appearing in the region δ 3.50–4.20 ppm. The ^13^C-NMR spectrum (Figure 3B) of PD1-1 showed typical fructan peaks according to previously reported values for fructans [21]. The anomeric carbons signals at 103.36, 104.90, and 92.61 ppm were attributed to C-2 of →1)-β-d-Fruf-(2→, →1)-β-d-Fruf-4AC-(2→, and C-1 of α-d-Glcp-(1→, respectively. C-1, C-3, C-4, C-5 and C-6 in →1)-β-d-Fruf-(2→ were assigned to signals at 61.10, 77.21, 74.51, 81.23, and 62.28 ppm, respectively. Signals appearing in a similar region were assigned to →1)-β-d-Fruf-(2→ residues.

In the HMBC spectrum, H-3 in →1)-β-d-Fruf-(2→ correlated with C-1 and C-4 in →1)-β-d-Fruf-4AC-(2→, while H-4 in →1)-β-d-Fruf-(2→ correlated with C-3, C-5, and C-6 in →1)-β-d-Fruf-4AC-(2→, confirming the presence of a →2-β-d-Fruf-1→ glycosidic linkage [22]. Furthermore, H-1 in α-d-Glcp-(1→ correlated with C-2 in →1)-β-d-Fruf-(2→, which confirmed that the glycosidic linkage of PD1-1 contained α-d-Glcp-1→2-β-D-Fruf-1→. C-2 correlated with H-1a and H-1b in →1)-β-d-Fruf-(2→, indicating that the →2)-β-d-Fruf-1→2-β-d-Fruf-(1→ glycosidic linkage was present. C-2 in →1)-β-d-Fruf-(2→ correlated with H-1b in →1)-β-d-Fruf-4AC-(2→, indicating that the →1)-β-d-Fruf-(2→1)-β-d-Fruf-4AC-(2→ linkage was present. In conclusion, PD1-1 is a fructan with a →2-β-d-Fruf-1→2-β-d-Fruf-1→ linkage and an acetyl group attached to the hydroxyl group at C-4 [23].

#### 2.3.4. Congo Red Test

**Figure 4:**
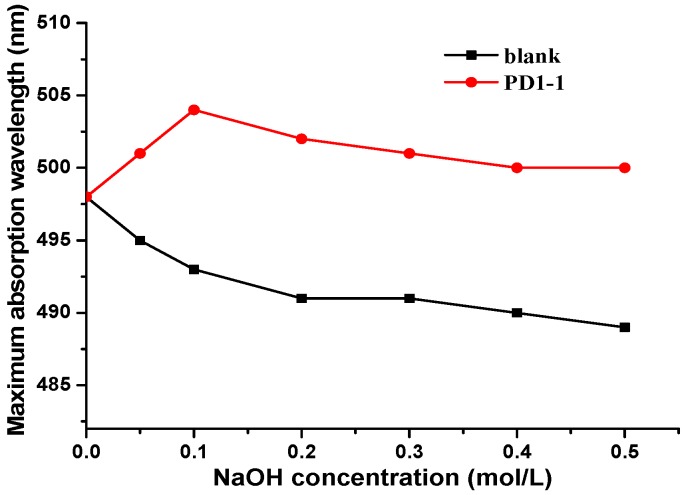
Maximum absorption plots of PD1-1 Congo red complex at various NaOH concentrations.

The triple-helix conformation of PD1-1 was analyzed using Congo red. According to previous reports, polysaccharides with triple helixes undergo a helix-coil transition with increasing alkali concentration, causing the maximum absorption wavelength (λ_max_) to be redshifted or blueshifted relative to pure Congo red [24]. As shown in Figure 4, with increasing NaOH concentration, the λ_max_ of Congo red combined with PD1-1 was redshifted. The highest value of λ_max_ was obtained when the NaOH concentration was 0.1 mol/L. Furthermore, the absorption decreased gradually with increasing NaOH concentration. This result indicates that PD1-1 exists as a triple helix structure.

### 2.4. Physicochemical Properties of PD1-1

The physicochemical properties of PD1-1 are shown in Table 3. PD1-1 is an odorless pale yellow powder soluble in water and insoluble in ethanol, methanol, and acetone. PD1-1 had the highest swelling capacity (SC) in distilled water, followed by phosphate buffer, with the lowest SC in 0.1 mol/L HCl, indicating that PD1-1 is sensitive to pH. The high SC of PD1-1 implies that the polymer could be used as a binder agent. The relatively higher water holding capacity (WHC) (9.57 ± 0.29 g/g) of PD1-1 compared to polysaccharides from potatoes peels [25] suggests that it could be used in many gourmet foods that require moisture. The pH value of PD1-1 was 6.47 ± 0.11, indicating that PD1-1 is a neutral sugar. The oil holding capacity (OHC) value of PD1-1 was calculated to be 4.36 ± 0.28 g/g. A similar OHC has been reported for potatoes peels (4.398 ± 0.04 g/g), suggesting that PD1-1 might be applied in the food industry. These results demonstrate that PD1-1 might be a suitable for use in the food and pharmaceutical industries.

### 2.5. Evaluation of Antioxidant Activities

**Figure 5:**
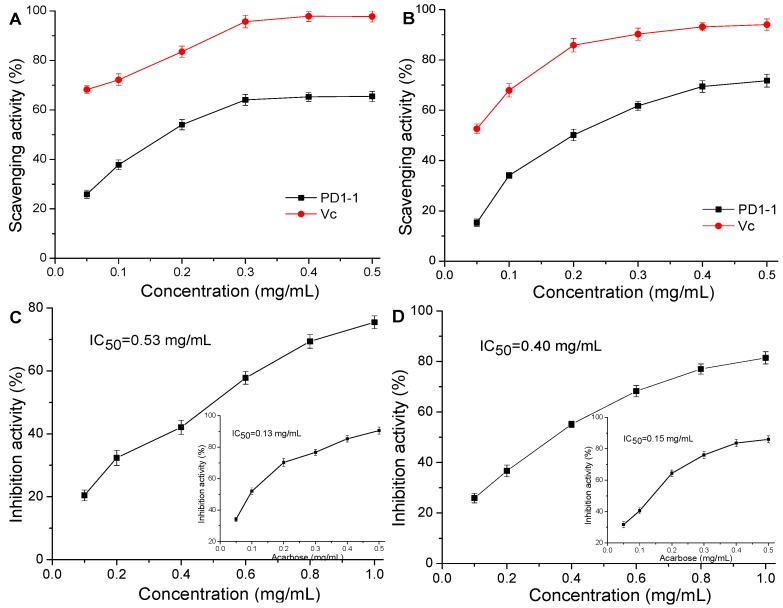
Antioxidant and hypoglycemic activities of PD1-1 in vitro. (**A**) DPPH radical scavenging activity; (**B**) hydroxyl radical scavenging activity; (**C**) α-glucosidase inhibitory activity; and (**D**) α-amylase inhibitory activity. Data presented as mean ± SD.

DPPH is an extremely stable free radical, while hydroxyl free radicals are extremely reactive. These radicals are commonly used to evaluate the free radical scavenging abilities of natural products [26,27]. The scavenging activity of PD1-1 and Vc toward DPPH radicals is shown in Figure 5A. Both PD1-1 and Vc showed dose-dependent scavenging abilities for DPPH radicals over the concentrations of 0.05–0.5 mg/mL. The scavenging rate increased significantly from 0.05–0.3 mg/mL, with the highest PD1-1 scavenging rate (65.48%) observed at 0.5 mg/mL. The half maximal inhibitory concentration (IC_50_) of PD1-1 was calculated to be 0.23 mg/mL, which was significantly higher than that of Vc. These results are consistent with previous research by Huang Dan [28], which showed that a polysaccharide from dandelion had a remarkable scavenging ability for DPPH, with an IC_50_ of 0.51 mg/mL. Therefore, PD1-1 showed a better scavenging ability than the above-mentioned polysaccharide. PD1-1 and Vc also showed hydroxyl radical scavenging abilities (Figure 5B) that were dose-dependent over the 0.05–0.5 mg/mL concentration range. The highest hydroxyl scavenging rate of PD1-1 (71.77%) was observed at 0.5 mg/mL and the IC_50_ of PD1-1 was calculated to be 0.25 mg/mL.

These results indicate that PD1-1 has strong antioxidant activity, although its free radical scavenging ratio was lower than that of Vc. In addition, the DPPH and hydroxyl radical scavenging abilities of the other two bathes of PD1-1 that were obtained by the same purifying method were also determined and achieved similar results, with IC_50_ values of 0.23 ± 0.03 mg/mL and 0.24 ± 0.02 mg/mL, respectively (Figures not shown). Previous studies [29,30] showed that the antioxidant ability of polysaccharides is closely related to their structure, physicochemical properties, and other components, such as proteins and uronic acid. Xing et al. [31] found that scavenging activity of a low molecular weight polysaccharide against a superoxide radical was higher than that of one with a high molecular weight. The specific association between structure, physicochemical properties, and activity requires further research.

### 2.6. α-Glucosidase and α-Amylase Inhibitory Activities

α-Amylase and α-glucosidase are the key enzymes that hydrolyze starch and other polysaccharides to monosaccharides and disaccharides in the small intestine. Furthermore, α-glucosidase inhibitors can retard the formation of glucose and treat type-2 pre-diabetic states. Therefore, α-glucosidase inhibition provides a prospective approach for preventing and treating type-2 diabetes [32,33]. The α-glucosidase inhibition activities of PD1-1 and acarbose are shown in Figure 5C. PD1-1 showed a dose-dependent inhibitory effect on α-glucosidase activity from 0.1 to 1.0 mg/mL. The IC_50_ of PD1-1 against α-glucosidase was calculated to be 0.53 mg/mL, which was higher than that of acarbose (0.13 mg/mL). The α-amylase inhibition activities of PD1-1 and acarbose are shown in Figure 5D. PD1-1 also showed a dose-dependent inhibitory effect on α-amylase activity from 0.1 to 1.0 mg/mL. The IC_50_ of PD1-1 against α-amylase was calculated to be 0.40 mg/mL, which was higher than that of acarbose (0.15 mg/mL). Although the IC_50_ of PD1-1 showed that it had a lower inhibitory activity than acarbose, PD1-1 still had a strong inhibitory effect on α-glucosidase and α-amylase. Furthermore, the α-glucosidase and α-amylase inhibitory activities of the other two batches of PD1-1 that were obtained by the same purifying method were also determined and achieved similar results, with IC_50_ values of 0.51 ± 0.02 mg/mL and 0.42 ± 0.01 mg/mL, respectively (Figures not shown). These results indicate that PD1-1 has potential as an antidiabetic drug.

## 3. Materials and Methods

### 3.1. Materials and Chemicals

The whole plant of dandelion was picked from Hami City in the Xinjiang Uygur Autonomous Region of China, and authenticated by Prof. Guogang Chen at Shihezi University. DEAE–Sepharose fast flow and Sephadex G-75 columns were purchased from Beijing Biotopped Science and Technology Co., Ltd. (Beijing, China). Standard dextrans, α-amylase, and α-glucosidase were all purchased from Sigma Chemical Co. (St. Louis, MO, USA). P-nitrophenyl-β-d-galactopyranoside (PNPG) was obtained from Shanghai Baomanbio Biotechnology Co., Ltd. (Shanghai, China). DPPH was purchased from the Tokyo Chemical Industry (Tokyo, Japan). All of the other chemical reagents used in this study were of analytical grade and from Xinjiang Tooken Biotechnology Co., Ltd. (Shihezi, Xinjiang, China). 

### 3.2. Extraction and Purification of Polysaccharides

Air-dried dandelion (1 kg) was pulverized and passed through a 60-mesh sieve. Fat and colored materials were removed using petroleum ether and 95% ethanol by refluxing, respectively. The residues were air-dried using a thermostatic blast drying oven at 50 °C. The pretreated dandelion powder was extracted with deionized water (1:25, *w*/*v*) and sonicated at 100 W and 75 °C for 65 min. All water extracts were combined, the suspension was filtrated under vacuum, and the supernatant was concentrated. Proteins were removed from the concentrated supernatant using the Sevag reagent [34]. The solution was precipitated with 4 volumes of 95% (*v*/*v*) ethanol for 12 h at 4 °C. The precipitate was collected by centrifugation and crude PD was obtained by vacuum freeze-drying.

Crude PD was fractionated using a DEAE–Sepharose fast flow column (10 × 60 cm). Crude PD (10 g) was dissolved in distilled water (50 mL), centrifuged, and transferred onto the column. Gradient elution was performed using distilled water followed by different concentrations of gradient NaCl solution (0.2, 0.5, 1.0, 2.0 mol/L) at 4 mL/min. The eluate was collected and elution peaks of the polysaccharides were monitored using a phenol sulfuric acid method (PSAM) at 490 nm [35]. The main fraction was pooled, desalted, concentrated, and further purified using a Sephadex G-75 column (2.6 × 60 cm) which was eluted with distilled water at a flow rate of 1 mL/min. The elution peaks were monitored by PSAM and the fraction was collected, denoted as PD1-1.

### 3.3. Chemical Analysis of PD1-1

#### 3.3.1. Chemical Component Analysis

The total sugar content was determined by PSAM using glucose as a standard. The protein content was determined using Bradford’s method [36], and the carbazole–sulfuric acid method [37] was used to measure the uronic acid content of PD1-1. 

#### 3.3.2. Determination of Molecular Weight

The molecular weight of PD1-1 was determined by HPGPC using a Shimadzu LC-10A system equipped with a Waters 2410 refractive index detector (Tosoh Corp., Tokyo, Japan) [38]. The mobile phase was double-distilled water (ddH_2_O) at a flow rate of 0.8 mL/min and a temperature of 40 °C. A 10 μL sample of PD1-1 solution (2.0 mg/mL) was injected into the chromatography system for each run. The molecular weight of PD1-1 was calculated by comparison with the calibration curve obtained from dextran standards (5.0–670 kDa).

#### 3.3.3. Monosaccharide Composition Analysis

The monosaccharide composition was determined by GC–MS following a previously reported procedure. The polysaccharide samples (2 mg) were dissolved in 2 mol/L trifluoracetic acid (TFA; 1 mL) in a closed tube. After hydrolysis at 110 °C for 90 min, the hydrolysate was dried in a vacuum evaporator and then dissolved in H_2_O (2 mL) containing NaBH_4_ (60 mg) at room temperature for 8 h. The evaporation step was then repeated under reduced pressure to remove the H_2_O after neutralization with glacial acetic acid. The samples were cooled and acetic anhydride (1 mL) was added. The mixture was incubated at 100 °C for 1 h and the evaporation step was then repeated under reduced pressure to remove the acetic anhydride with toluene (3 mL). The acetylated hydrolysate was extracted with chloroform and dried by anhydrous sodium sulfate. The monosaccharide composition was analyzed by GC–MS and identified according to characteristic retention times. Mannose, rhamnose, fucose, xylose, glucose, galactose, and arabinose were used as monosaccharide standards.

### 3.4. Structural Analysis of PD1-1

#### 3.4.1. UV and FT-IR Spectrometric Analysis

PD1-1 polysaccharide was dissolved in distilled water to a final concentration of 5% and analyzed using UV–vis spectrophotometry. The UV absorption spectrum of the sample was recorded in the 200–400 nm wavelength range. FT-IR spectra of PD1-1 were recorded using an FT-IR spectrometer by mixing the sample (2 mg) thoroughly with KBr powder (100 mg) and pressing into a disk. The FT-IR spectrum of PD1-1 was recorded in the 400–4000 cm^−1^ frequency range.

#### 3.4.2. Methylation Analysis

Methylation analysis of PD1-1 was conducted according to a previously reported method [39]. Briefly, PD1-1 was dissolved in anhydrous dimethyl sulfoxide (DMSO; 1 mL), and solid NaOH was added to the solution under a nitrogen atmosphere. The sample was then methylated with CH_3_I, and the permethylated sample was hydrolyzed with 2 mol/L TFA at 121 °C for 1.5 h. After reduction and acetylation of the hydrolysates, the sample was analyzed by GC–MS using an RXI-5 SIL MS column.

#### 3.4.3. NMR Analysis

PD1-1 (30 mg) was dissolved in 99.9% D_2_O (0.6 mL). 1D and 2D NMR spectra were recorded using an AV-500 spectrometer equipped with a 5-mm CPQCI 1H-31P/13C/15N/D Z-GRD cryoprobe with samples in 5-mm NMR tubes [40]. Spectra were obtained at 25 °C using 120 scans. Data processing was performed using standard Bruker Topspin-NMR software (AVANCE III, Bruker Corp., Fällanden, Switzerland).

#### 3.4.4. Congo Red Test

The Congo red test was performed according to a previously reported method with slight modifications [41]. First, the sample solution (1 mL, 2 mg/mL) was mixed with a NaOH solution (1 mL; 0, 0.1, 0.2, 0.4, 0.6, 0.8, or 1 mol/L). This process afforded NaOH solutions with final concentrations of 0, 0.1, 0.2, 0.3, 0.4, and 0.5 mol/L. Next, Congo red (2 mL, 80 μmol) was added and mixed thoroughly. A sample solution without NaOH was used as reference. Furthermore, the maximum absorption wavelength was scanned using a UV-2600 spectrophotometer (Shimadzu Corp., Kyoto, Japan) in the 200–600 nm wavelength range after incubating for 10 min at room temperature.

### 3.5. Physicochemical Properties of PD1-1

#### 3.5.1. Determination of Solubility and pH

The solubility of PD1-1 was measured in water, ethanol, methanol, and acetone according to the British Pharmacopoeia (BP) specification [42]. The pH of PD1-1 in distilled water at a concentration of 1% (*w*/*v*) was determined using a PHS-3C pH meter.

#### 3.5.2. Determination of Hydration Properties

The WHC was determined using a previously reported method [43]. Briefly, the initial volume (*V*_1_) occupied by PD1-1 (500 mg) in a test tube was recorded. Next, distilled water (10 mL) was added, the mixture was allowed to stand for 12 h at room temperature, and the new volume (*V*_2_) of the wetted powder was recorded. This test was repeated using a phosphate buffer (pH 6.86) and 0.1 mol/L HCl instead of distilled water. The SC was calculated using the following formula: SC (%) = (*V*_2_ − *V*_1_)/*V*_1_ × 100.

The WHC was determined simultaneously. PD1-1 (500 mg, *M*_0_) was dispersed in distilled water (10 mL), stirred, kept at room temperature for 1 h, and centrifuged at 3000 rpm for 10 min. The supernatant was removed, the residue was weighed (*M*_1_), and the WHC was calculated using the following formula: WHC (g/g) = (*M*_1_ − *M*_0_)/*M*_0_.

#### 3.5.3. Oil Holding Capacity

The OHC was determined using the method described above for WHC determination, but using soybean oil instead of distilled water. The OHC was calculated as g of soybean oil retained per g of sample on a dry basis.

### 3.6. Antioxidant Activities of Polysaccharides

#### 3.6.1. DPPH Radical Scavenging Activity

The DPPH radical scavenging activity of PD was determined using a previously reported method with some modifications [44]. Briefly, a DPPH solution (2.0 mL, 0.1 mmol/L in ethanol) was mixed with a PD solution (2.0 mL) at various concentrations. After mixing rapidly, the mixture was incubated at room temperature for 30 min in the dark, and the absorbance was measured at 517 nm. Distilled water was used as the blank control, and vitamin C (Vc) was used as the positive control. The DPPH radical scavenging activity (RSA) was calculated according to the following equation:

DPPH RSA (%) = [1 − (*A*_2_ − *A*_1_)/*A*_0_] × 100% (1)

where *A*_2_ is the absorbance of a mixture of the sample and the DPPH solution, *A*_1_ is the absorbance of the sample without the DPPH solution, and *A*_0_ is the absorbance of the DPPH solution without the sample.

#### 3.6.2. Hydroxyl Radical Scavenging Activity

The hydroxyl radical scavenging activity of PD was estimated using a previously reported Fenton-type reaction with some modifications [45]. Sample solutions (1 mL) of various concentrations were mixed with FeSO_4_ (1 mL, 9 mmol/L), salicylic acid (1 mL, 9 mmol/L), and H_2_O_2_ (1 mL, 9 mmol/L) solutions. The mixtures were incubated at 37 °C for 30 min and the absorbance was measured at 510 nm. Distilled water was used as the blank control and Vc was used as the positive control. The hydroxyl radical scavenging activity was calculated according to the following equation:

Hydroxyl RSA (%) = [1 − (*A*_2_ − *A*_1_)/*A*_0_] × 100% (2)

where *A*_2_ is the absorbance of a mixture of the sample and the H_2_O_2_ solution, *A*_1_ is the absorbance of the sample without the H_2_O_2_ solution, and *A*_0_ is the absorbance of the H_2_O_2_ solution without the sample.

### 3.7. Hypoglycemic Activity Assay

#### 3.7.1. α-Glucosidase Inhibition Assay

The α-glucosidase inhibitory activity was determined using the method reported by Zhang with some modifications [46]. Briefly, the reaction mixture containing a phosphate buffer (2 mL, 0.1 mol/L, pH 6.8), α-glucosidase (0.1 mL, 0.2 U/mL), and the sample solution (1 mL) was incubated at 37 °C for 15 min. P-nitrophenyl-β-d-galactopyranoside (PNPG; 250 μL) was then added, followed by further incubation at 37 °C for 30 min. The catalytic reaction was terminated by adding Na_2_CO_3_ solution (2 mL, 0.1 mol/L). Acarbose was used as the positive control and the absorbance was measured at 400 nm. The α-glucosidase inhibition rate was calculated using the following equation:

α-Glucosidase inhibition rate (%) = [1 − (*A*_2_ − *A*_1_)/*A*_0_] × 100% (3)

where *A*_2_ is the absorbance of the sample solution, *A*_1_ is the absorbance of the PNPG and sample solution without adding the enzyme, and *A*_0_ is the absorbance of PNPG solution with the enzyme and without the sample.

#### 3.7.2. α-Amylase Inhibition Assay

α-amylase inhibitory activity was determined using the method reported by Wang with slight modifications [8]. Briefly, sample solutions (1 mL) were mixed with α-amylase (1 mL, 1.0 U/mL) and incubated for 15 min at 37 °C. A starch solution (1 mL, 1%) was then added, followed by incubation for a further 10 min at 37 °C. The enzyme reaction was stopped by adding 3,5-dinitrosalicylic acid (DNS; 2 mL) while heating over a boiling water bath for 5 min. The reaction mixture was diluted by adding distilled water and the absorbance was measured at 540 nm. Acarbose was used as the positive control. The α-amylase inhibition rate was calculated using the following equation:

α-Amylase inhibition rate (%) = [1 − (*A*_2_ − *A*_1_)/*A*_0_] × 100% (4)

where *A*_2_ is the absorbance of the sample solution, *A*_1_ is the absorbance of the starch and sample solution without adding the enzyme, and *A*_0_ is the absorbance of the starch solution with the enzyme and without adding the sample.

### 3.8. Statistical Analysis

All experiments were conducted at least in triplicate and values are expressed as means ± standard deviation (SD). Statistical analyses were performed using SPSS 19.0 statistical software (SPSS Inc., Chicago, IL, USA).

## 4. Conclusions

In the present study, a crude polysaccharide from dandelion was prepared using an ultrasound-assisted extraction method, with one purified fraction (PD1-1) obtained by chromatography using DEAE–Sepharose fast flow and Sephadex G-75 columns. The monosaccharide composition showed that PD1-1 is an inulin-type polysaccharide with glucose (52.39%) and mannose (45.41%) as the main components. Molecular weight analysis showed that PD1-1 has a molecular weight of 2.6 kDa. Glycosidic linkage analysis showed that PD1-1 contains α-d-Man/Glcp-(1→ and →1)-β-d-Man/Glcf-(2→ glycosidic linkage conformations. Furthermore, PD1-1 has a triple-helix structure and exhibits important physicochemical properties, including good SC, WHC, and OHC values. Furthermore, the in vitro antioxidant and hypoglycemic activities of PD1-1 were determined, showing strong biological activities that are concentration-dependent. Therefore, we predict that PD1-1 could be widely applied in the pharmaceutical and food industries. However, the in vivo functional properties of PD1-1 are still unclear, and research into its in vivo structure–activity relationship is ongoing on.

## Figures and Tables

**Table 1:** PD1-1 methylation results.

Methylated Sugars	Linkage Type	Molar Ratio (%)	Major Mass Fragments (*m*/*z*)
2,3,4,6-Me_4_-Glc/Manp	Terminal Man/Glc	2	45, 71, 87, 101, 117, 129, 145, 161, 205
3,4,6-Me_3_-Glc/Manf	1,2-linked Man/Glc	14	43, 87, 129, 161, 187

**Table 2:** ^1^H and ^13^C nuclear magnetic resonance (NMR) chemical shifts (ppm) for PD1-1.

Glycosyl Residues	H1a/H1b	H2	H3	H4	H5	H6a/H6b
	C1	C2	C3	C4	C5	C6
→1)-β-D-Fruf-(2→	3.68/3.87	-	4.21	4.06	3.82	3.80/3.73
	61.10	103.36	77.21	74.51	81.23	62.28
α-D-Glcp-1→	5.40	3.51	3.71	4.01	4.22	3.82
	92.61	71.31	72.79	69.43	73.70	75.68
→1)-β-D-Fruf-4AC-(2→	3.77/3.62	-	4.21	4.17	4.08	3.77/3.62
	61.42	104.9	78.56	81.45	83.10	64.60

**Table 3:** Physicochemical properties of PD1-1.

Parameters	Results
Solubility	Soluble in water, insoluble in ethanol, methanol, and acetone
Swelling capacity (%, *v*/*v*):	
distilled water	201 ± 5.56
HCl (0.1 mol/L)	159 ± 4.17
Phosphate buffer (pH 6.86)	185 ± 4.82
pH	6.47 ± 0.11
water holding capacity (WHC) (g/g)	9.57 ± 0.29
oil holding capacity (OHC) (g/g)	4.36 ± 0.28

## Supplementary Materials

The following are available online, Figure S1, The calibration curve of dextran standards and HPGPC of TMP-1-1 polysaccharide. Figure S2, GC-MS diagram of monosaccharide standard and TMP-1-1.
