# Supplementary material for: Physicochemical, Structural, and Biological Properties of Polysaccharides from Dandelion
Source: Molecules. 2019 Apr 15;24(8):1485. doi: 10.3390/molecules24081485 (PMC6514733; doi:10.3390/molecules24081485)
Supplement: Supplementary file 1 [file molecules-24-01485-s001.pdf]

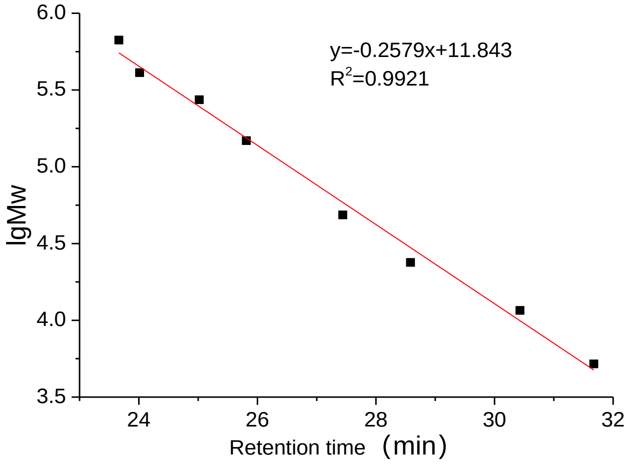
y=-0.2579x+11.843
R2=0.9921
lgMw
6.0
5.5
5.0
4.5
4.0
3.5
24
26
28
30
32
Retention time （min）

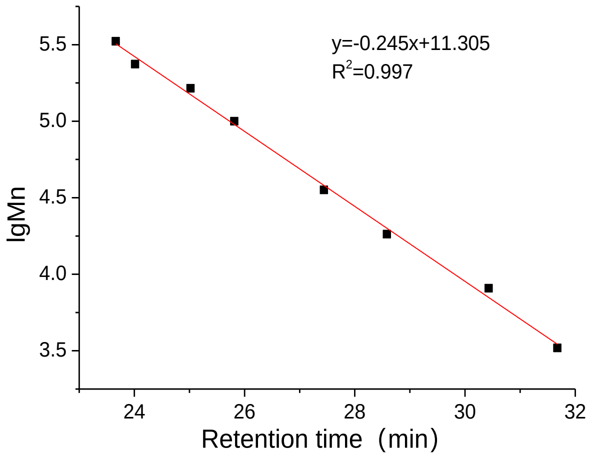
y=-0.245x+11.305
R2=0.997
lgMn
5.5
5.0
4.5
4.0
3.5
24
26
28
30
32
Retention time （min）

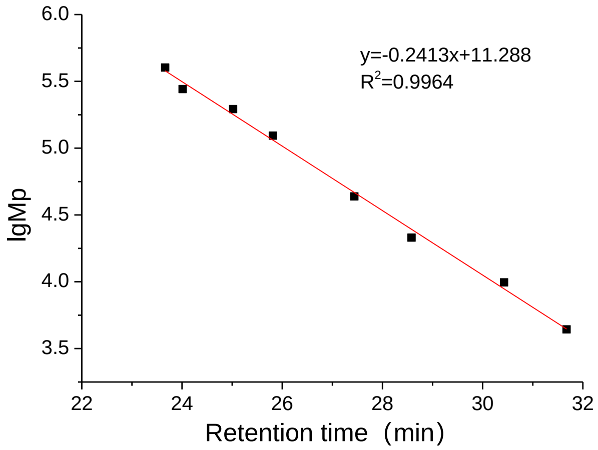
y=-0.2413x+11.288
R2=0.9964
lgMp
6.0
5.5
5.0
4.5
4.0
3.5
22
24
26
28
30
32
Retention time （min）

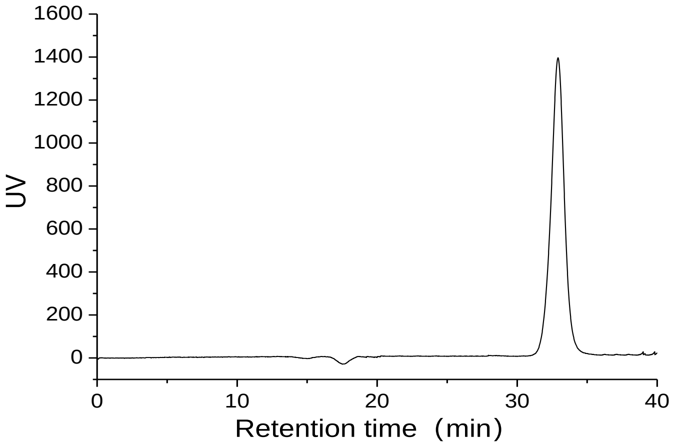

Figure.S1. The calibration curve of dextran standards and HPGPC of TMP-1-1 polysaccharide

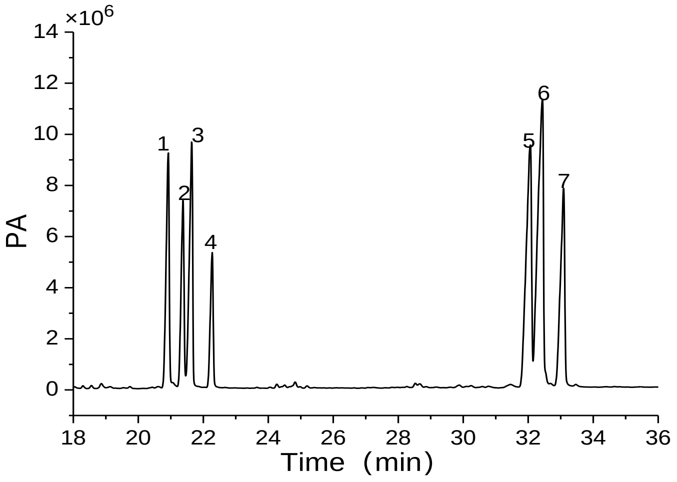

Note: Peak 1-rhamnose, 2-fucose, 3-arabinose, 4-xylose, 5-mannose, 6-glucose, 7-galactose

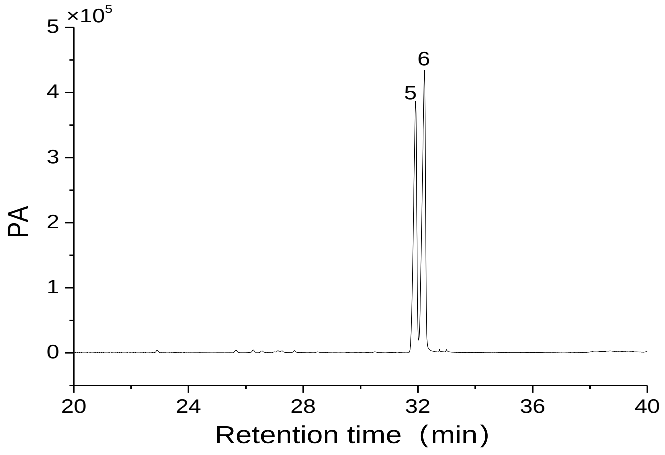

Figure.S2. GC-MS diagram of monosaccharide standard and TMP-1-1
